# Digital Technologies in Implantology: A Narrative Review

**DOI:** 10.3390/bioengineering12090927

**Published:** 2025-08-29

**Authors:** Ani Kafedzhieva, Angelina Vlahova, Bozhana Chuchulska

**Affiliations:** Department of Prosthetic Dentistry, Faculty of Dental Medicine, Medical University of Plovdiv, 4000 Plovdiv, Bulgaria; angelina.vlahova@mu-plovdiv.bg (A.V.); bozhana.chuchulska@mu-plovdiv.bg (B.C.)

**Keywords:** artificial intelligence, bioengineering, CAD/CAM technologies, digital implantology, guided surgery

## Abstract

Digital technologies have significantly advanced implant dentistry, refining diagnosis, treatment planning, surgical precision, and prosthetic rehabilitation. This review explores recent developments, emphasizing accuracy, efficiency, and clinical impact. A literature analysis identifies key innovations, such as digital planning, guided surgery, dynamic navigation, digital impressions and CAD/CAM prosthetics. Digital workflows enhance implant placement by improving precision and reducing deviations compared to freehand techniques. Dynamic navigation provides real-time guidance, offering accuracy comparable to static guides and proving benefits in complex cases. Digital impressions demonstrate high precision, which can match or, in some scenarios, surpass conventional methods, though conventional impressions remain the gold standard for full-arch cases. CAD/CAM technology optimizes prosthetic fit, aesthetics, and material selection. Artificial intelligence and machine learning contribute to treatment planning and predictive analytics, yet challenges persist, including high costs, the need for specialized training, and long-term clinical validation. This review underscores the advantages of digital approaches—improved accuracy, better communication, and minimally invasive procedures—while addressing existing limitations. Emerging technologies, such as AI, augmented reality, and 3D printing, are expected to further transform implantology. Continued research is crucial to fully integrate digital advancements and enhance patient outcomes.

## 1. Introduction

Implant dentistry has undergone a significant transformation with the integration of digital technologies over the past decade. These advancements have revolutionized various aspects of implant treatment, from diagnosis and planning to surgical execution and prosthetic rehabilitation [1]. Dental implants have become a widely accepted treatment option for replacing missing teeth, offering improved function, aesthetics, and quality of life for patients [2].

The evolution of digital technologies in dentistry has been rapid and transformative. From the introduction of cone-beam computed tomography (CBCT) for 3D imaging to the development of sophisticated computer-aided design and computer-aided manufacturing (CAD/CAM) systems, the field has seen a paradigm shift in how implant treatments are planned and executed [3]. This digital revolution has not only enhanced the precision and predictability of implant procedures but has also opened new avenues for patient communication and treatment customization [4].

The integration of digital workflows in implantology has been driven by several factors. Firstly, the increasing demand for aesthetic and functional outcomes in implant dentistry has necessitated more precise and predictable treatment modalities [5]. Secondly, the growing complexity of cases, including immediate implant placement and full-arch rehabilitations, has highlighted the limitations of traditional techniques and the potential benefits of digital approaches [6]. Thirdly, the rapid advancement of computer technology and software capabilities has made sophisticated digital tools more accessible and user-friendly for clinicians [7].

Building upon these technological advances, the integration of bioengineering principles with artificial intelligence enables sophisticated biomechanical analysis and predictive modeling that optimize treatment outcomes. This multidisciplinary approach facilitates digital workflows encompassing real-time stress monitoring and advanced biomaterial selection, advancing toward more personalized and evidence-based implant therapy.

Digital technologies in implantology encompass a wide range of applications, including:

1. Three-dimensional imaging and diagnostics using CBCT and intraoral scanning;
2. Virtual implant planning and surgical guide design;
3. Computer-guided and dynamic navigation surgery;
4. Digital impression techniques for implant-supported restorations;
5. CAD/CAM fabrication of implant prosthetics;
6. Artificial intelligence and machine learning applications for treatment planning and outcome prediction.

Each of these areas has seen significant advancements in recent years, with numerous studies demonstrating improvements in accuracy, efficiency, and patient outcomes compared to conventional techniques [8,9,10].

However, the adoption of digital technologies in implantology is not without challenges. The initial investment in equipment and software can be substantial, and there is a learning curve associated with implementing new digital workflows [11]. Additionally, concerns have been raised about the long-term reliability and potential limitations of digital techniques in certain clinical scenarios [12].

The aim of this narrative review is to provide a comprehensive analysis of the current state of digital technologies in implantology, evaluating their applications, benefits, and limitations. Specifically, this review aims to:

1. Evaluate the current applications of digital technologies in implantology
2. Assess the accuracy and efficacy of digital methods compared to conventional approaches
3. Identify the benefits and limitations of digital workflows in implant dentistry
4. Explore future perspectives and emerging trends in digital implantology

By synthesizing the latest research and clinical evidence, this review seeks to provide clinicians, researchers, and industry professionals with a thorough understanding of the current landscape of digital implantology and its potential future directions. This descriptive analysis will help inform clinical decision-making, guide future research efforts, and contribute to the ongoing evolution of digital technologies in implant dentistry.

## 2. Materials and Methods

This narrative review aims to provide a descriptive overview of current developments in digital technologies applied in implantology. A targeted literature search was conducted across major scientific databases, including PubMed, Web of Science, Scopus, and Embase. Relevant publications were identified using keywords such as “digital implantology”, “guided implant surgery”, “dynamic navigation”, “digital impressions”, “CAD/CAM in implantology”, “artificial intelligence in implantology”, and “3D printing in implant dentistry”.

The selection of sources was based on their relevance to the main themes of the review, with a focus on recent advancements, clinical applications, and future trends. No formal inclusion or exclusion criteria or standardized quality assessment tools were applied as the objective was to synthesize and summarize key concepts and innovations rather than perform a systematic evaluation. In total, 73 publications were included to support the narrative discussion.

## 3. Results

### 3.1. Digital Planning and Guided Surgery

**Figure 1:**
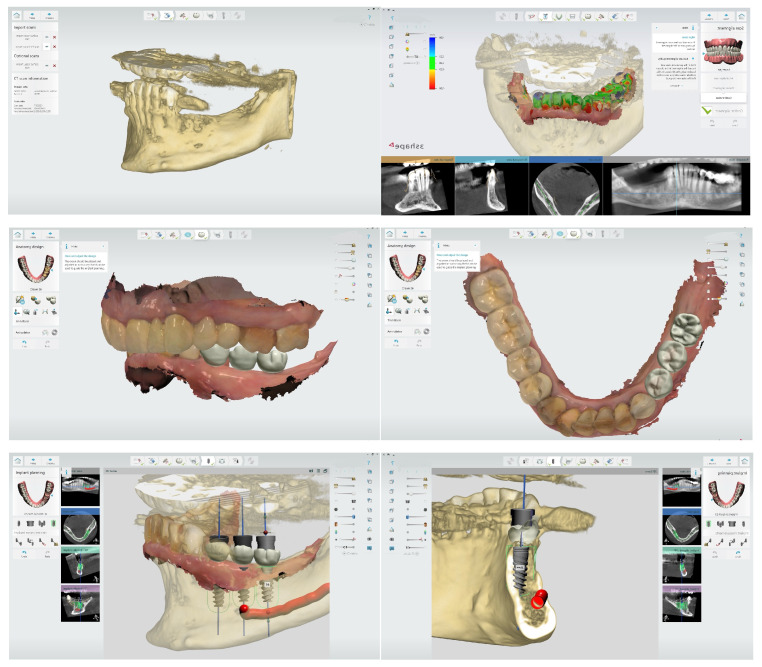
Virtual planning of the optimal prosthetic position of dental implants using 3Shape software. The digital workflow includes alignment of the intraoral scan with the CBCT data, followed by prosthetically driven implant positioning based on the final restoration design. 3Shape Implant Studio 2021.2. CAD/CAM Center, Faculty of Dental Medicine, Medical University of Plovdiv.

Cone-beam computed tomography (CBCT) has revolutionized implant planning by providing high-resolution, three-dimensional images of the patient’s anatomy. This technology allows for precise evaluation of bone quality, quantity, and the position of vital structures, enabling more accurate implant placement [13]. Advanced software packages integrate CBCT data with intraoral scans or digital impressions to create comprehensive treatment plans. These tools allow clinicians to virtually place implants, considering both anatomical and prosthetic requirements (Figure 1) [14].

A preliminary study by Fonseca et al. evaluated implant placement accuracy in 53 implants using pilot drill guided and fully guided approaches, revealing an average angular deviation of 3.90° and mean linear deviations of 1.04 mm at the coronal point and 1.56 mm at the apex [15]. This improved accuracy can lead to better implant positioning and reduced risk of complications. A study by Rodrigues et al. demonstrated that guided surgery techniques resulted in significantly lower angular and linear deviations compared to the conventional technique, confirming their superior accuracy in implant placement [16].

The integration of bioengineering methods in implant planning has opened new avenues for personalized treatment approaches. By combining radiographic data with three-dimensional files, clinicians can now predict and evaluate potential rehabilitation outcomes with unprecedented accuracy [17]. This not only enhances the predictability of the treatment but also allows for optimization of implant placement based on biomechanical principles [18].

Recent advancements in digital planning software have introduced machine learning algorithms to assist in optimal implant positioning. These algorithms analyze vast databases of successful implant cases to suggest ideal implant locations based on patient-specific anatomy and prosthetic requirements [19]. Furthermore, the integration of augmented reality (AR) in surgical planning allows clinicians to visualize virtual implants superimposed on the patient’s actual anatomy, enhancing spatial understanding and treatment communication [20].

A prospective clinical study by Younis et al. evaluated the accuracy of implant placement using dynamic navigation, static surgical guides, and freehand techniques, revealing mean angular deviations of 3.66°, 2.52°, and 5.82°, respectively, with both computer-assisted methods demonstrating significantly greater precision than freehand placement [21]. Additionally, a study by Sarkar et al. found that guided surgery significantly reduced operative time and post-operative pain compared to conventional techniques [22].

A long-term follow-up study by Naeini et al. on static computer-aided implant surgery (sCAIP) reported a 97.1% implant survival rate over an average of 9.1 years, with a mean bone loss of 0.63 mm. While peri-implant health parameters were generally favorable, 6.3% of implants developed peri-implantitis, and 43.8% of patients experienced technical complications. Despite these findings, sCAIP demonstrated comparable long-term clinical outcomes to conventional, non-guided implant surgery [23].

Regarding the materials used for printed surgical guides, Paradowska-Stolarz et al. report that BioMed Amber resin is commonly applied for the fabrication of strong and rigid surgical guides for dental implants, where precise placement is critical. Their study found that the highest compressive modulus (0.88 ± 0.03 GPa) and tensile modulus (2.88 ± 0.26 GPa) were observed in specimens that were neither polished nor artificially aged, while both polishing and artificial aging significantly reduced these mechanical properties, which is essential to consider when preparing surgical guides for implantology applications [24].

Digital planning for orthodontic mini-implant placement utilizes advanced 3D imaging, virtual planning, and CAD/CAM technologies to optimize miniscrew positioning, minimize complications, and improve accuracy—an approach that closely parallels the established digital workflows in implantology [25]. In the study by Wilmes, which details the Beneslider system methodology, a digital approach is also described, highlighting the integration of virtual planning and CAD/CAM-fabricated guides for precise and efficient appliance placement [26].

**Figure 2:**
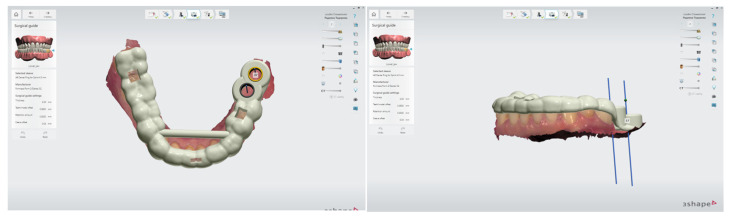
Static surgical guide designed for guided implant surgery. The guide is digitally planned based on CBCT data and intraoral scans, allowing for precise three-dimensional positioning of the implants according to the prosthetically driven treatment plan. 3Shape Implant Studio. CAD/CAM Center, Faculty of Dental Medicine, Medical University of Plovdiv.

Digital technologies enable the precise planning and fabrication of static surgical guides, which offer numerous advantages including improved accuracy of implant placement, reduced surgical time, enhanced patient safety, and greater predictability of clinical outcomes (Figure 2).

### 3.2. Dynamic Navigation Systems

Dynamic navigation systems provide real-time guidance during implant surgery, allowing for adjustments based on the patient’s actual position and movement. This technology offers flexibility and the potential for minimally invasive procedures [27]. A meta-analysis by Pellegrino et al. of 32 studies assessing dynamic navigation for implant placement found mean entry and apical deviations of 0.81 mm and 0.91 mm, respectively, with an angular deviation of 3.81°. Dynamic navigation demonstrated significantly higher accuracy than freehand placement (*p* < 0.01) and comparable precision to static guides (*p* ≥ 0.05), with a low implant failure rate of 1%, supporting its reliability as an accurate implant placement method [28].

The advent of dynamic navigation has significantly impacted the field of implantology by providing surgeons with real-time feedback and guidance during the procedure. This technology allows for immediate adjustments based on the patient’s anatomy and movement, potentially reducing the risk of errors and improving overall surgical outcomes [29]. Moreover, dynamic navigation systems have shown promise in complex cases in which traditional static guides may be challenging to use, such as in patients with limited mouth opening or in immediate implant placement scenarios [30].

A meta-analysis of 10 studies evaluating dynamic computer-assisted implant surgery found mean global platform, apex, and angular deviations of 1.02 mm, 1.33 mm, and 3.59°, respectively, with no significant differences based on study design, jaw location, or implant system, confirming its clinically acceptable accuracy and potential for widespread use [31]. Furthermore, a study on dynamic navigation-assisted implant placement by Ma et al. reported mean apex, tip, and angle deviations of 1.60 mm, 1.83 mm, and 3.80°, respectively, with greater tip deviation in anterior regions and a significant reduction in angle deviation with surgical experience, confirming its superior accuracy over traditional methods [32].

Recent innovations in dynamic navigation technology include the integration of haptic feedback systems, providing tactile sensations to surgeons during implant placement. This advancement aims to enhance the surgeon’s ability to detect subtle changes in bone density and improve the precision of osteotomy preparation [33]. Additionally, the development of markerless tracking systems has simplified the workflow of dynamic navigation, eliminating the need for fiducial markers and potentially reducing patient discomfort [34].

The learning curve associated with dynamic navigation systems has been a topic of interest. A study by Spille et al. evaluating the learning curve of young professionals using dynamic navigation for implant placement found significant improvements in angular and apex deviations (*p* < 0.001) over 160 placements, demonstrating that the technique can be quickly mastered and effectively applied in clinical practice [35].

### 3.3. Digital Impressions

**Figure 3:**
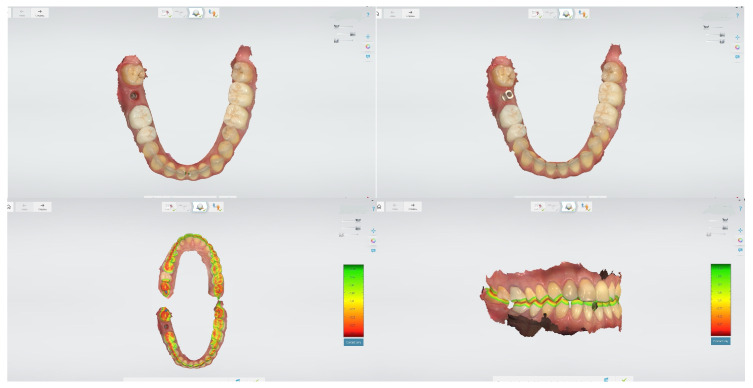
Intraoral scan demonstrating a digital impression of an implant site using a scan body. The scan body is accurately positioned onto the implant to transfer the three-dimensional spatial orientation, enabling precise alignment of the virtual implant model within the CAD software for prosthetic design. 3Shape TRIOS 2. CAD/CAM Center, Faculty of Dental Medicine, Medical University of Plovdiv.

Digital impressions have emerged as a game-changing technology in implant dentistry, offering numerous advantages over conventional impression techniques. Intraoral scanners capture detailed three-dimensional images of the oral cavity, eliminating the need for traditional impression materials and providing immediate visualization of the scanned data (Figure 3) [36].

#### 3.3.1. Accuracy and Precision

A systematic review and meta-analysis by Papaspyridakos et al. concluded that digital impressions show comparable or better accuracy than conventional methods for partial restorations [37]. The study found that digital techniques offer advantages in terms of time efficiency, patient comfort, and ease of use. For single-implant cases, digital impressions showed a mean 3D deviation of 8.20 µm in fully edentulous patients and 52.31 µm in partially edentulous patients, with conventional impressions being slightly more accurate (*p* = 0.03) [38].

The accuracy of digital impressions for full-arch implant cases has been a topic of extensive research. While earlier studies showed limitations in full-arch scans, recent technological advancements have significantly improved their performance. The accuracy of three intraoral scanners was evaluated, showing that prefabricated aids improved trueness and precision, reducing RMS errors from 67.5 to 61.8 μm (IOS-T), 100.6 to 45.9 μm (IOS-M), and 52.7 to 41.1 μm (IOS-A), with IOS-A and IOS-M demonstrating the highest precision and a strong correlation between scan errors and prosthetic misfit [39].

Similarly, a clinical study by Arikan et al. confirmed the importance of scan accuracy for implant-supported full-arch prostheses, demonstrating that the highest mean vertical marginal gap (80.86 ± 50.06 μm) occurred with intraoral scanner impressions alone, while the lowest (41.98 ± 26.33 μm) was found in the control group. The study further supported the findings from citation 37 by showing that auxiliary geometric appliances improved digital scan accuracy, reinforcing the notion that additional aids enhance trueness and precision in digital impressions. Conventional splinted open-tray impressions still provided the most reliable fit, suggesting that, while digital methods are improving, they require optimization for full-arch cases [40].

A study by Emam et al. found that the Medit i500 (Medit Corp., Seoul, Republic of Korea) intraoral scanner achieved the highest trueness for digital impressions of post spaces, with a mean root mean square (RMS) deviation of 0.18 ± 0.03 mm for 8 mm depths and 0.18 ± 0.06 mm for 10 mm depths, followed by Primescan AC (Dentsply Sirona, Bensheim, Germany) (0.31 ± 0.07 mm for 8 mm and 0.20 ± 0.07 mm for 10 mm), while CS 3600 (Carestream Dental, Atlanta, GA, USA) showed the lowest trueness (0.33 ± 0.09 mm for 8 mm and 0.26 ± 0.11 mm for 10 mm). These results indicate that, for implant-related scanning procedures requiring accurate post-space capture, Medit i500 and Primescan AC are preferable, as CS 3600 not only had lower trueness but also failed to reliably capture the full post-space depth, especially at 10 mm [41].

Modern intraoral scanners employ the same structured-light projection and photogrammetric reconstruction principles demonstrated by Martins et al. in their 12 MP laboratory optical scanner protocol, which yielded model deviations below 20 µm; intraoral devices are thus capable of delivering clinical trueness on the order of 20–50 µm, meeting the accuracy demands of digital impressions [42].

#### 3.3.2. Workflow Efficiency and Patient Experience

One of the key advantages of digital impressions in implant dentistry is the ability to capture the implant position immediately after placement. This allows for the fabrication of immediate provisional restorations with high accuracy, improving patient satisfaction and treatment efficiency [43]. A meta-analysis by de Oliveira et al. shows that digital workflow for single implant crowns demonstrated superior clinical efficiency compared to the conventional workflow, with significantly reduced impression time (MD: 8.22 min), higher patient preference, and improved time efficiency, while adjustment time showed variable results across studies [44].

Moreover, digital workflows enable the seamless integration of implant planning data with prosthetic design, facilitating more predictable and aesthetically pleasing outcomes [45]. A study by Schubert et al. demonstrated that combining CBCT data, intraoral scans, and CAD/CAM fabrication enhances precision, safety, and efficiency by allowing accurate backward planning for optimal implant positioning. This integrated approach improves functional, biological, and aesthetic predictability while minimizing surgical risks. However, potential inaccuracies in manufacturing and application must be carefully managed to prevent complications [46].

The adoption of digital impression techniques has not only improved the accuracy of implant prosthetics but has also streamlined the workflow between clinicians and dental laboratories. The ability to instantly transmit digital files has reduced turnaround times and minimized the risk of errors associated with traditional impression materials and shipping [47]. A systematic review and meta-analysis by Bessadet et al. further confirmed the efficiency of digital workflows, demonstrating significantly reduced impression and laboratory times compared to conventional and hybrid methods (*p* < 0.05), as well as lower laboratory costs. While adjustment times showed no significant differences, digital workflows consistently optimized time efficiency and resource utilization in implant-supported prosthesis fabrication [48].

Additionally, digital impressions allow for easy storage and retrieval of patient data, facilitating long-term follow-up and simplifying the process of creating replacement prostheses when needed [49]. A systematic review by Ahmed et al. analyzed the benefits of digital impression. The study found that digital impression techniques demonstrate superior efficiency and time savings compared to conventional methods, significantly reducing procedure duration and streamlining the workflow, as supported by clinical studies [50].

#### 3.3.3. Technological Advancements and Innovations

Recent innovations in digital impression technology include the development of artificial intelligence-assisted scanning protocols. These systems provide real-time feedback to clinicians during the scanning process, ensuring complete coverage and highlighting areas that require rescanning [51]. A narrative review by Altalhi et al. evaluated AI and found that it is revolutionizing implant dentistry by enhancing diagnostics, treatment planning, and patient outcomes through advanced image analysis, deep learning, and data-driven precision [52].

Furthermore, the integration of color and texture information in digital scans has enhanced the ability to capture soft tissue details [53]. A study by Mahato et al. indicates that digital workflows provide comparable or superior accuracy in clinical marginal fit, occlusion, and interproximal contacts, while also offering greater patient comfort and potentially more precise and well-adapted final restorations compared to conventional methods [54].

#### 3.3.4. Challenges and Limitations

Despite the numerous advantages, challenges remain in certain clinical scenarios. Subgingival implant margins and deep soft tissue pockets can be difficult to capture accurately with intraoral scanners. To address this, new scanning protocols and specialized scan bodies have been developed to improve the accuracy of digital impressions in these challenging situations [55]. A study by Wu et al. investigates how different scanbody exposure heights affect the trueness and precision of digital implant impressions in anterior and posterior regions. The findings reveal that exposures below 6 mm in the anterior and 4 mm in the posterior lead to increased deviations, though still within clinically acceptable limits [56].

Digital impressions in full-arch implant rehabilitations of atrophied maxillae have been reported to exhibit greater two- and three-dimensional deviations when compared to conventional splinted open-tray impression techniques. These deviations may contribute to a higher incidence of nonpassive frameworks and misfit restorations, potentially affecting the long-term success and stability of the prosthesis. Therefore, in complex edentulous cases with multiple implants and challenging anatomy, conventional impression techniques may provide more consistent and reliable accuracy [57].

Another challenge in digital impressions is the learning curve associated with mastering the scanning technique. The in vivo study by Roth et al. evaluated the learning curve of intraoral scanning by analyzing changes in scanning time and image count among dental students. The results demonstrated a significant reduction in scanning time with experience, while image count initially decreased but later increased after the sixth measurement, indicating a need for corrections to maintain scan quality [58].

#### 3.3.5. Future Directions

The future of digital impressions in implantology looks promising, with ongoing research focusing on improving scanning technologies and expanding their applications. Emerging technologies such as photogrammetry and light-field imaging are being explored as potential alternatives to current scanning methods, offering the possibility of even greater accuracy and ease of use [59].

A clinical report by Azvedo et al. presents a digital workflow combining photogrammetry and conventional impressions to enhance the accuracy of implant-supported screw-retained prostheses. Five patients were treated using a superimposed digital model, leading to a CAD/CAM-fabricated prosthesis with improved fit, function, and aesthetics, though further studies are needed to validate photogrammetry’s accuracy compared to conventional techniques [60].

### 3.4. CAD/CAM in Implant Prosthetics

**Figure 4:**
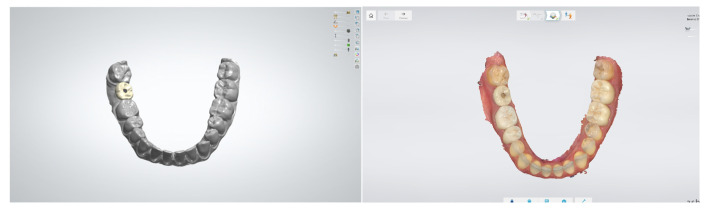
Digital workflow for an implant-supported restoration. The image illustrates the virtual design of the crown in 3Shape CAD software (**left**) and the final screw-retained crown in situ (**right**). The use of digital technologies ensures accurate prosthetic planning, optimal fit, and streamlined clinical procedures. 3Shape Dental System. CAD/CAM Center, Faculty of Dental Medicine, Medical University of Plovdiv.

Computer-aided design and computer-aided manufacturing (CAD/CAM) technologies have revolutionized the fabrication of implant-supported prostheses. These technologies offer improved fit, reduced production time, and the ability to work with a wider range of materials [61]. CAD software for implant prosthetics has evolved to offer more intuitive interfaces and advanced design capabilities. These tools allow for the creation of highly customized restorations that consider both functional and aesthetic requirements (Figure 4) [62].

Advancements in manufacturing technologies have expanded the range of materials and production methods available for implant prosthetics. Computer-aided manufacturing (CAM) systems, including milling machines and 3D printers, can produce highly accurate and consistent restorations [63]. The range of materials available for CAD/CAM implant prosthetics has expanded significantly, including high-strength ceramics, hybrid materials, and biocompatible metals. Rexhepi et al. reviewed the mechanical and aesthetic properties of various CAD/CAM materials in restorative and prosthetic dentistry, highlighting the importance of material selection based on specific clinical requirements. The authors summarize that zirconia is the primary material for implant-supported restorations due to its exceptional strength, durability, and aesthetic properties, making it suitable for crowns, bridges, and full-arch rehabilitations. Additionally, PEEK, PICN, and nanoceramics serve as alternative solutions, with PEEK excelling in implant abutments and bar-supported prostheses, while PICN and nanoceramics offer improved aesthetics and mechanical properties for implant-supported crowns and bridges [64]. Building on these developments, recent research highlights the promising role of advanced polymeric biomaterials, particularly polyetheretherketone (PEEK) and its composites, in improving the mechanical performance and biocompatibility of dental implants and abutments. A comprehensive review by Hanna et al. synthesizes current findings on these materials, emphasizing their biomechanical behavior and potential to enhance implant longevity [65].

A recent innovation in CAD/CAM implant prosthetics is the development of “one-abutment, one-time” protocols. This approach involves the design and fabrication of a final abutment immediately after implant placement, which remains in place throughout the healing and restoration process. Studies have shown that this technique can lead to better preservation of peri-implant soft tissues and improved aesthetic outcomes, but with no statistical difference [66].

The integration of CAD/CAM technologies with digital impression systems has created a fully digital workflow for implant prosthetics. This seamless process allows for greater precision in the design and fabrication of restorations, as well as improved communication between clinicians and dental laboratories [67]. A study by Corsalini et al. found that fully digital workflows resulted in significantly better marginal fit of implant-supported crowns compared to conventional techniques [68].

### 3.5. Artificial Intelligence and Machine Learning in Implantology

Artificial intelligence (AI) algorithms are being developed to assist in implant treatment planning by analyzing CBCT data and suggesting optimal implant positions. These tools have the potential to enhance decision-making and improve treatment outcomes [69]. Machine learning models are being used to predict implant success rates based on patient-specific factors and treatment parameters. Nazari et al. discussed the integration of AI and machine learning in implant dentistry workflows, highlighting their potential to improve risk assessment and treatment customization [70].

For instance, Ahn et al. demonstrated that a parametric reduced-order model based on finite element–derived stress data can rank key implant placement factors by their biomechanical impact and drive a digital twin framework for real-time evaluation of implant plans. This approach offers clinicians objective, data-driven insights into stress distribution patterns, enhancing precision and predictability in implant therapy [71].

AI-powered decision support systems are emerging as valuable tools for clinicians, providing evidence-based recommendations for implant selection, treatment planning, and prosthetic design. These systems can analyze vast amounts of data from previous cases, scientific literature, and clinical guidelines to offer personalized treatment suggestions [72].

A study by Alqutaibi et al. demonstrated the effectiveness of an AI algorithm in detecting and classifying peri-implant bone loss on radiographs. The AI system achieved a sensitivity and specificity ranged from 67% to 95% and 78% to 100%, respectively, outperforming general dentists and matching the performance of experienced implantologists [73]. This suggests that AI could serve as a valuable tool for early detection of implant complications and support ongoing patient monitoring.

## 4. Discussion

The integration of digital technologies in implantology has led to a profound transformation of clinical workflows, with substantial improvements in diagnostic precision, surgical accuracy, and prosthetic outcomes. Three-dimensional imaging modalities, such as cone-beam computed tomography (CBCT), have become essential for preoperative assessment, enabling detailed visualization of bone structures and vital anatomical features. When combined with intraoral scanning and advanced planning software, CBCT data allows for virtual implant placement that accounts for both anatomical constraints and prosthetic requirements, supporting a truly prosthetically driven approach [13,14]. Clinical studies have demonstrated that static guided surgery, based on this digital planning, significantly improves the accuracy of implant positioning compared to conventional freehand techniques, as evidenced by reduced angular and linear deviations [15,16]. This enhanced precision not only optimizes implant placement but also minimizes the risk of complications, particularly in challenging cases with limited bone availability or complex anatomical situations [16,73].

The evolution of digital planning has also seen the introduction of bioengineering methods and machine learning algorithms, which facilitate personalized treatment strategies and predictive analytics. By leveraging large datasets and computational models, clinicians can optimize implant positioning and anticipate potential outcomes, further increasing the predictability of implant therapy [17,18,19]. Augmented reality applications are emerging as adjuncts to digital planning, providing enhanced visualization and communication tools for both clinicians and patients [20].

Dynamic navigation systems have emerged as a significant advancement, offering real-time, intraoperative guidance that allows for immediate adjustments based on patient movement and anatomical variations. Meta-analyses confirm that dynamic navigation achieves accuracy levels comparable to static guides and superior to freehand approaches, with mean entry and apical deviations typically below 1 mm and angular deviations around 3.8° [28,31]. These systems are particularly valuable in scenarios where static guides are impractical, such as immediate implant placement or cases with restricted access [30,32]. Technological innovations, including haptic feedback and markerless tracking, have further streamlined dynamic navigation workflows, while studies indicate that clinicians can rapidly acquire proficiency with these systems, making them accessible and effective in routine practice [33,34,35].

Digital impression techniques, utilizing intraoral scanners, have revolutionized the capture of implant positions and soft tissue morphology. Systematic reviews and meta-analyses show that digital impressions offer accuracy comparable to or exceeding that of conventional methods for single and partial restorations, with notable advantages in patient comfort and workflow efficiency [37,44]. For full-arch rehabilitations, recent advancements—such as auxiliary geometric devices and improved scanning protocols—have significantly enhanced the trueness and precision of digital impressions, though conventional splinted open-tray techniques may still provide superior accuracy in complex edentulous cases [39,40,57]. The ability to instantly transmit digital files to dental laboratories reduces turnaround times and minimizes potential errors, while digital records facilitate long-term follow-up and simplify the fabrication of replacement prostheses [47,49]. However, challenges remain in capturing subgingival margins and deep soft tissue pockets, necessitating ongoing development of specialized scan bodies and scanning protocols [55,56].

The adoption of digital workflows has also led to measurable improvements in clinical efficiency and patient-reported outcomes. Studies demonstrate that digital impression techniques reduce procedure times, enhance patient satisfaction, and streamline communication between clinicians and laboratories [44,48]. The learning curve associated with intraoral scanning is manageable, with significant reductions in scanning time and improvements in scan quality observed as clinicians gain experience [58].

Digital workflows, while often associated with higher initial investment in equipment and software, have been shown to be cost-effective over the long term by reducing operative time, minimizing material waste, and decreasing the need for remakes and adjustments—thereby lowering overall treatment costs and improving practice profitability. Furthermore, the ability to capture, store, and reuse digital implant data streamlines recalls and restorative maintenance, translating into ongoing savings for both practices and patients.

CAD/CAM technologies have fundamentally changed the design and fabrication of implant-supported prostheses. Modern CAD software enables the creation of highly customized restorations that integrate functional and aesthetic considerations, while advanced manufacturing methods, such as milling and 3D printing, provide restorations with exceptional fit and consistency [61,62,63]. The range of materials available for CAD/CAM prosthetics has expanded to include high-strength ceramics like zirconia, as well as hybrid materials and biocompatible polymers, allowing for tailored solutions based on specific clinical requirements [64]. Innovations such as the “one-abutment, one-time” protocol support soft tissue preservation and improved aesthetic outcomes, although long-term studies are needed to confirm their superiority [66]. Fully digital workflows, integrating digital impressions and CAD/CAM fabrication, have been shown to produce restorations with better marginal fit and higher patient satisfaction compared to conventional methods [67,68].

Artificial intelligence and machine learning are increasingly being applied in implantology, assisting with treatment planning, risk assessment, and outcome prediction. AI algorithms can analyze CBCT data and patient-specific factors to suggest optimal implant positions and predict success rates, supporting evidence-based clinical decision-making [69,70,72]. AI-powered diagnostic tools have demonstrated high sensitivity and specificity in detecting peri-implant bone loss, outperforming general dentists and matching experienced implantologists [73]. These technologies hold promise for early detection of complications and ongoing patient monitoring.

Despite these advances, several challenges persist. The initial cost of digital equipment and software can be substantial, and there is a learning curve for clinicians adopting new workflows. While digital methods offer clear advantages in many scenarios, conventional techniques may still be preferable in certain complex cases, such as full-arch rehabilitations with multiple implants and challenging anatomy [57]. Long-term clinical outcomes of fully digital workflows require further validation, and the lack of standardized protocols can lead to inconsistencies in treatment outcomes. Data security and patient privacy are also critical considerations as digital data management becomes more prevalent.

The rapid evolution of digital technologies in implantology underscores the need for dental education curricula and continuing professional development programs to integrate hands-on training in CAD/CAM systems, intraoral scanning, and dynamic navigation. Embedding these competencies into undergraduate and postgraduate courses, as well as offering modular online and in-person CPD workshops, will ensure that clinicians remain proficient with emerging tools, ultimately enhancing patient care and fostering lifelong learning.

In summary, digital technologies have reshaped implantology by improving the accuracy, efficiency, and predictability of implant treatments. The evidence supports the superiority of digital workflows in many aspects, including surgical planning, impression-taking, and prosthetic fabrication, while also highlighting the need for ongoing research, clinical validation, and the development of standardized protocols. As digital tools continue to evolve and become more accessible, their integration into routine practice is expected to further enhance patient outcomes and satisfaction, shaping the future of implant dentistry.

## 5. Conclusions

Digital technologies have significantly transformed the field of implantology, offering improved accuracy, efficiency, and predictability in implant treatments. This narrative review has highlighted the advancements in various aspects of digital implantology, including digital planning and guided surgery, dynamic navigation systems, digital impressions, CAD/CAM in implant prosthetics, and the integration of artificial intelligence and machine learning.

The evidence presented in this review demonstrates that digital workflows can lead to more precise implant placement, reduced surgical time, and improved prosthetic outcomes. Digital impressions, in particular, have shown comparable or superior accuracy to conventional methods, while offering additional benefits in terms of patient comfort, workflow efficiency, and long-term data management.

However, the adoption of digital technologies in implantology is not without challenges. The initial investment costs, learning curve for clinicians, and the need for long-term clinical data remain important considerations. Additionally, while digital technologies offer numerous advantages, they should be viewed as tools to complement clinical expertise rather than replace it entirely.

As the field of digital implantology continues to evolve, future research should focus on the following:

1. Long-term clinical outcomes and biological effects of fully digital workflows, especially in complex cases and full-arch rehabilitations, where robust longitudinal data are still lacking.
2. The clinical accuracy, reliability, and practical implementation of emerging technologies such as photogrammetry, light-field imaging, and augmented reality for implant planning and impression-taking.
3. The standardization and reproducibility of digital workflows across different software platforms, hardware systems, and operator experience levels, to ensure consistent clinical results.
4. The integration and autonomous application of artificial intelligence and machine learning algorithms in routine clinical decision-making, risk assessment, and treatment outcome prediction.
5. The impact of digital implantology on peri-implant tissue health, and the incidence of biological and technical complications over extended follow-up periods.
6. Patient-reported outcomes, satisfaction, and psychological acceptance of digital implant treatments compared to conventional protocols, particularly in diverse populations and complex cases.
7. Data security, privacy, and ethical management of digital patient information within fully digital implantology workflows.

In conclusion, digital technologies in implantology offer promising solutions to enhance treatment outcomes and patient care. As these technologies continue to mature and become more integrated into routine clinical practice, they are likely to play an increasingly central role in shaping the future of dental implantology. Clinicians are encouraged to stay informed about these advancements and consider incorporating digital workflows into their practice to provide optimal care for their patients.

## References

[1] Joda T., Zarone F., Ferrari M. (2017). The complete digital workflow in fixed prosthodontics: A systematic review. BMC Oral Health.

[2] Buser D., Sennerby L., De Bruyn H. (2017). Modern implant dentistry based on osseointegration: 50 years of progress, current trends and open questions. Periodontol. 2000.

[3] Mangano F., Shibli J.A., Fortin T. (2016). Digital Dentistry: New Materials and Techniques. Int. J. Dent..

[4] Lee S.J., Jamjoom F.Z., Le T., Radics A., Gallucci G.O. (2022). A clinical study comparing digital scanning and conventional impression making for implant-supported prostheses: A crossover clinical trial. J. Prosthet. Dent..

[5] Att W., Witkowski S., Strub J.R. (2019). Digital workflow in reconstructive dentistry. Quintessence Int..

[6] Pozzi A., Arcuri L., Moy P.K. (2018). The smiling scan technique: Facially driven guided surgery and prosthetics. J. Prosthodont. Res..

[7] Khaohoen A., Powcharoen W., Sornsuwan T., Chaijareenont P., Rungsiyakull C., Rungsiyakull P. (2024). Accuracy of implant placement with computer-aided static, dynamic, and robot-assisted surgery: A systematic review and meta-analysis of clinical trials. BMC Oral Health..

[8] Joda T., Brägger U., Gallucci G. (2015). Systematic literature review of digital three-dimensional superimposition techniques to create virtual dental patients. Int. J. Oral Maxillofac. Implant..

[9] Mangano F., Veronesi G. (2018). Digital versus Analog Procedures for the Prosthetic Restoration of Single Implants: A Randomized Controlled Trial with 1 Year of Follow-Up. Biomed. Res. Int..

[10] Patzelt S.B.M., Spies B.C., Kohal R.J. (2015). CAD/CAM-fabricated implant-supported restorations: A systematic review. Clin. Oral Implant. Res..

[11] Joda T., Gallucci G.O. (2015). The virtual patient in dental medicine. Clin. Oral Implant. Res..

[12] Pozzi A., Tallarico M., Moy P.K. (2014). Three-year post-loading results of a randomised, controlled, split-mouth trial comparing implants with different prosthetic interfaces and design in partially posterior edentulous mandibles. Eur. J. Oral Implantol..

[13] Jacobs R., Salmon B., Codari M., Hassan B., Bornstein M.M. (2018). Cone beam computed tomography in implant dentistry: Recommendations for clinical use. BMC Oral Health.

[14] Tahmaseb A., Wu V., Wismeijer D., Coucke W., Evans C. (2018). The accuracy of static computer-aided implant surgery: A systematic review and meta-analysis. Clin. Oral Implant. Res..

[15] Fonseca C.M., da Fonseca P.A.B., Quezada M.M., Marques T., Montero J., Morton D., Correia A. (2024). Analyzing Linear and Angular Deviations After Guided Surgery for Dental Implant Placement: A Preliminary Study. Int. J. Oral Maxillofac. Implant..

[16] Rodrigues J.M.M., Santos P.L., Mendonça G., Faloni A.P.S., Finoti L.S., Margonar R. (2023). Assessment of Deviations of Implants Installed with Prototyped Surgical Guide and Conventional Guide: In Vitro Study. Eur. J. Dent..

[17] Joda T., Zitzmann N.U. (2022). Personalized workflows in reconstructive dentistry-current possibilities and future opportunities. Clin. Oral Investig..

[18] Lin P., Su K. (2020). Biomechanical Design Application on the Effect of Different Occlusion Conditions on Dental Implants with Different Positions—A Finite Element Analysis. Appl. Sci..

[19] Alharbi M.T., Almutiq M.M. (2022). Prediction of Dental Implants Using Machine Learning Algorithms. J. Healthc. Eng..

[20] Mai H.N., Dam V.V., Lee D.H. (2023). Accuracy of Augmented Reality-Assisted Navigation in Dental Implant Surgery: Systematic Review and Meta-analysis. J. Med. Internet Res..

[21] Younis H., Lv C., Xu B., Zhou H., Du L., Liao L., Zhao N., Long W., Elayah S.A., Chang X. (2024). Accuracy of dynamic navigation compared to static surgical guides and the freehand approach in implant placement: A prospective clinical study. Head. Face Med..

[22] Sarkar A., Hoda M.M., Malick R., Kumar A. (2022). Surgical Stent Guided Versus Conventional Method of Implant Placement. J. Maxillofac. Oral Surg..

[23] Naeini E.N., De Bruyn H., Bronkhorst E.M., D’haese J. (2023). Long-Term Effect of Guided Implant Surgery on Clinical Outcomes and Peri-Implantitis of Maxillary Implants-An Observational Cohort Study. J. Clin. Med..

[24] Paradowska-Stolarz A., Mikulewicz M., Wieckiewicz M., Wezgowiec J. (2023). The Influence of Polishing and Artificial Aging on BioMed Amber^®^ Resin’s Mechanical Properties. J. Funct. Biomater..

[25] Akdeniz B.S., Çarpar Y., Çarpar K.A. (2022). Digital three-dimensional planning of orthodontic miniscrew anchorage: A literature review. J. Exp. Clin. Med..

[26] Wilmes B. (2023). The new Benefit for Aligner Technique to overcome limitations of aligners. J. Aligner Orthod..

[27] Mahmoud N.R., Kamal Eldin M.H., Diab M.H., Mahmoud O.S., Fekry Y.E. (2024). Computer guided versus freehand dental implant surgery: Randomized controlled clinical trial. Saudi Dent. J..

[28] Pellegrino G., Ferri A., Del Fabbro M., Prati C., Gandolfi M.G., Marchetti C. (2021). Dynamic Navigation in Implant Dentistry: A Systematic Review and Meta-analysis. Int. J. Oral Maxillofac. Implant..

[29] Zinser M., Neugebauer J., Mischkowski R., Karapedian V., Kübler A., Zöller J. (2004). Comparison of static and dynamic navigation systems for insertion of dental implants. Int. Congr. Ser..

[30] Wang F., Fan S., Huang W., Shen Y., Li C., Wu Y. (2022). Dynamic navigation for prosthetically driven zygomatic implant placement in extensive maxillary defects: Results of a prospective case series. Clin. Implant. Dent. Relat. Res..

[31] Wei S.M., Zhu Y., Wei J.X., Zhang C.N., Shi J.Y., Lai H.C. (2021). Accuracy of dynamic navigation in implant surgery: A systematic review and meta-analysis. Clin. Oral Implant. Res..

[32] Ma L., Ye M., Wu M., Chen X., Shen S. (2023). A retrospective study of dynamic navigation system-assisted implant placement. BMC Oral Health.

[33] Bhalerao A., Ayoub A., Usman A. (2024). Application of Dynamic Navigation, Virtual Reality, and Universal Robot in Dental Implantology. Indian J. Dent. Res..

[34] Jorba-García A., Bara-Casaus J.J., Camps-Font O., Figueiredo R., Valmaseda-Castellón E. (2024). The influence of radiographic marker registration versus a markerless trace registration method on the implant placement accuracy achieved by dynamic computer-assisted implant surgery. An in-vitro study. J. Dent..

[35] Spille J., Helmstetter E., Kübel P., Weitkamp J.T., Wagner J., Wieker H., Naujokat H., Flörke C., Wiltfang J., Gülses A. (2022). Learning Curve and Comparison of Dynamic Implant Placement Accuracy Using a Navigation System in Young Professionals. Dent. J..

[36] Mangano F., Gandolfi A., Luongo G., Logozzo S. (2017). Intraoral scanners in dentistry: A review of the current literature. BMC Oral Health.

[37] Papaspyridakos P., Vazouras K., Chen Y.W., Kotina E., Natto Z., Kang K., Chochlidakis K. (2020). Digital vs Conventional Implant Impressions: A Systematic Review and Meta-Analysis. J. Prosthodont..

[38] Gianfreda F., Pesce P., Marcano E., Pistilli V., Bollero P., Canullo L. (2022). Clinical Outcome of Fully Digital Workflow for Single-Implant-Supported Crowns: A Retrospective Clinical Study. Dent. J..

[39] Fu X.J., Liu M., Shi J.Y., Deng K., Lai H.C., Gu W., Zhang X.M. (2025). Comparison of Different Intraoral Scanners with Prefabricated Aid on Accuracy and Framework Passive Fit of Digital Complete-Arch Implant Impression: An In Vitro Study. Clin. Oral Implant. Res..

[40] Arikan H., Muhtarogullari M., Uzel S.M., Guncu M.B., Aktas G., Marshall L.S., Turkyilmaz I. (2023). Accuracy of digital impressions for implant-supported complete-arch prosthesis when using an auxiliary geometry device. J. Dent. Sci..

[41] Emam M., Ghanem L., Abdel Sadek H.M. (2024). Effect of different intraoral scanners and post-space depths on the trueness of digital impressions. Dent. Med. Probl..

[42] Martins J.N.R., Pinto R., Silva E.J.N.L., Simões-Carvalho M., Marques D., Martins R.F., Versiani M.A. (2023). 3D Surface Scanning—A Novel Protocol to Characterize Virtual Nickel–Titanium Endodontic Instruments. Materials.

[43] Deeb J.G., Reddy N.G., Hopfensperger L.J., Harris A.L., Bencharit S. (2023). Same-Day Digital Dentistry Restorative Workflow for Single Immediate Provisionalization of Narrow-Diameter Implants: An Exploratory Prospective Study. Prosthesis.

[44] de Oliveira N.R.C., Pigozzo M.N., Sesma N., Laganá D.C. (2020). Clinical efficiency and patient preference of digital and conventional workflow for single implant crowns using immediate and regular digital impression: A meta-analysis. Clin. Oral Implant. Res..

[45] Flügge T., Kramer J., Nelson K., Nahles S., Kernen F. (2022). Digital implantology—A review of virtual planning software for guided implant surgery. Part. II: Prosthetic set-up and virtual implant planning. BMC Oral Health.

[46] Schubert O., Schweiger J., Stimmelmayr M., Nold E., Güth J.F. (2019). Digital implant planning and guided implant surgery–workflow and reliability. Br. Dent. J..

[47] Binhuraib H., Alreshidi F., Bardi S., Alghamdi N., Alhelali S., Althagafi T., Alsayegh R., Alsalem F., Aljathnan A., Alshahrani N. (2023). Evaluating the efficiency of complete digital workflow in prosthodontics. J. Healthc. Sci..

[48] Bessadet M., Auduc C., Drancourt N., Nicolas E., El Osta N. (2024). Comparative analyses of time efficiency and cost in fabricating fixed implant-supported prostheses in digital, hybrid, and conventional workflows: A systematic review and meta-analysis. J. Prosthet. Dent..

[49] Rutkūnas V., Gečiauskaitė A., Jegelevičius D., Vaitiekūnas M. (2017). Accuracy of digital implant impressions with intraoral scanners. A systematic review. . Eur. J. Oral Implantol..

[50] Ahmed S., Hawsah A., Rustom R., Alamri A., Althomairy S., Alenezi M., Shaker S., Alrawsaa F., Althumairy A., Alteraigi A. (2024). Digital Impressions Versus Conventional Impressions in Prosthodontics: A Systematic Review. Cureus.

[51] Panahi O. (2024). Dental Implants & the Rise of AI. On J. Dent. Oral Health.

[52] Altalhi A.M., Alharbi F.S., Alhodaithy M.A., Almarshedy B.S., Al-Saaib M.Y., Al Jfshar R.M., Aljohani A.S., Alshareef A.H., Muhayya M., Al-Harbi N.H. (2023). The Impact of Artificial Intelligence on Dental Implantology: A Narrative Review. Cureus.

[53] Marques S., Ribeiro P., Falcão C., Lemos B.F., Ríos-Carrasco B., Ríos-Santos J.V., Herrero-Climent M. (2021). Digital Impressions in Implant Dentistry: A Literature Review. Int. J. Environ. Res. Public. Health.

[54] Mahato M., Hota S., Jain A., Dutta D., Bhushan P., Raut A. (2024). Comparison of Conventional and Digital Workflows in the Fabrication of Fixed Prostheses: A Systematic Review. Cureus.

[55] Mizumoto R.M., Yilmaz B. (2018). Intraoral scan bodies in implant dentistry: A systematic review. J. Prosthet. Dent..

[56] Wu H.K., Chen G., Huang X., Deng F., Li Y. (2023). Accuracy of single-implant digital impression with various scanbody exposure levels at anterior and posterior regions. J. Dent..

[57] Jasim A.G., Abo Elezz M.G., Altonbary G.Y., Elsyad M.A. (2024). Accuracy of digital and conventional implant-level impression techniques for maxillary full-arch screw-retained prosthesis: A crossover randomized trial. Clin. Implant. Dent. Relat. Res..

[58] Róth I., Hermann P., Vitai V., Joós-Kovács G.L., Géczi Z., Borbély J. (2023). Comparison of the learning curve of intraoral scanning with two different intraoral scanners based on scanning time. BMC Oral Health.

[59] Peñarrocha-Oltra D., Agustín-Panadero R., Bagán L., Giménez B., Peñarrocha M. (2014). Impression of multiple implants using photogrammetry: Description of technique and case presentation. Med. Oral Patol. Oral Cir. Bucal.

[60] Azevedo L., Molinero-Mourelle P., Antonaya J.L., Río J., Correia A., Gomez-Polo M. (2019). Photogrammetry Technique for the 3D Digital Impression of Multiple Dental Implants. VipIMAGE 2019.

[61] Joda T., Ferrari M., Gallucci G.O., Wittneben J.G., Brägger U. (2017). Digital technology in fixed implant prosthodontics. Periodontol. 2000.

[62] Smith D.G., Burgess E.M. (2001). The use of CAD/CAM technology in prosthetics and orthotics--current clinical models and a view to the future. J. Rehabil. Res. Dev..

[63] Cai H., Xu X., Lu X., Zhao M., Jia Q., Jiang H.B., Kwon J.S. (2023). Dental Materials Applied to 3D and 4D Printing Technologies: A Review. Polymers.

[64] Rexhepi I., Santilli M., D’Addazio G., Tafuri G., Manciocchi E., Caputi S., Sinjari B. (2023). Clinical Applications and Mechanical Properties of CAD-CAM Materials in Restorative and Prosthetic Dentistry: A Systematic Review. J. Funct. Biomater..

[65] Hanna E.G., Amine S., Prasad B., Younes K. (2024). Exploring polyetheretherketone in dental implants and abutments: A focus on biomechanics and finite element methods. Rev. Adv. Mater. Sci..

[66] Sanz-Sánchez I., Molina A., Martin C., Bollain J., Calatrava J., Sanz M. (2024). The effect of one-time abutment placement on clinical and radiographic outcomes: A 5-year randomized clinical trial. Clin. Oral Implant. Res..

[67] Bernauer S.A., Zitzmann N.U., Joda T. (2023). The Complete Digital Workflow in Fixed Prosthodontics Updated: A Systematic Review. Healthcare.

[68] Corsalini M., Barile G., Ranieri F., Morea E., Corsalini T., Capodiferro S., Palumbo R.R. (2024). Comparison between Conventional and Digital Workflow in Implant Prosthetic Rehabilitation: A Randomized Controlled Trial. J. Funct. Biomater..

[69] Revilla-León M., Gómez-Polo M., Vyas S., Barmak B.A., Galluci G.O., Att W., Krishnamurthy V.R. (2023). Artificial intelligence applications in implant dentistry: A systematic review. J. Prosthet. Dent..

[70] Nazari Y., Lngeroodi P.F., Maddahi M., Kobravi S., Amin M.R., Bargrizaneh A.A., Fouladi S. (2025). Artificial intelligence models and predicting implant success. Biomed. Res. Ther..

[71] Ahn S., Kim J., Baek S., Kim C., Jang H., Lee S. (2024). Toward Digital Twin Development for Implant Placement Planning Using a Parametric Reduced-Order Model. Bioengineering.

[72] Chen Y.W., Stanley K., Att W. (2020). Artificial intelligence in dentistry: Current applications and future perspectives. Quintessence Int..

[73] Alqutaibi A.Y., Algabri R.S., Alamri A.S., Alhazmi L.S., Almadani S.M., Alturkistani A.M., Almutairi A.G. (2024). Advancements of artificial intelligence algorithms in predicting dental implant prognosis from radiographic images: A systematic review. J. Prosthet. Dent..

